# Lateralization of Eye Use in Cuttlefish: Opposite Direction for Anti-Predatory and Predatory Behaviors

**DOI:** 10.3389/fphys.2016.00620

**Published:** 2016-12-12

**Authors:** Alexandra K. Schnell, Roger T. Hanlon, Aïcha Benkada, Christelle Jozet-Alves

**Affiliations:** ^1^Normandie University (UNICAEN)Caen, France; ^2^Program in Sensory Physiology and Behavior, Marine Biological Laboratory (MBL)Woods Hole, MA, USA

**Keywords:** eye use, functional lateralization, cephalopod, invertebrate, brain specialization, vision

## Abstract

Vertebrates with laterally placed eyes typically exhibit preferential eye use for ecological activities such as scanning for predators or prey. Processing visual information predominately through the left or right visual field has been associated with specialized function of the left and right brain. Lateralized vertebrates often share a general pattern of lateralized brain function at the population level, whereby the left hemisphere controls routine behaviors and the right hemisphere controls emergency responses. Recent studies have shown evidence of preferential eye use in some invertebrates, but whether the visual fields are predominately associated with specific ecological activities remains untested. We used the European common cuttlefish, *Sepia officinalis*, to investigate whether the visual field they use is the same, or different, during anti-predatory, and predatory behavior. To test for lateralization of anti-predatory behavior, individual cuttlefish were placed in a new environment with opaque walls, thereby obliging them to choose which eye to orient away from the opaque wall to scan for potential predators (i.e., vigilant scanning). To test for lateralization of predatory behavior, individual cuttlefish were placed in the apex of an isosceles triangular arena and presented with two shrimp in opposite vertexes, thus requiring the cuttlefish to choose between attacking a prey item to the left or to the right of them. Cuttlefish were significantly more likely to favor the left visual field to scan for potential predators and the right visual field for prey attack. Moreover, individual cuttlefish that were leftward directed for vigilant scanning were predominately rightward directed for prey attack. Lateralized individuals also showed faster decision-making when presented with prey simultaneously. Cuttlefish appear to have opposite directions of lateralization for anti-predatory and predatory behavior, suggesting that there is functional specialization of each optic lobe (i.e., brain structures implicated in visual processing). These results are discussed in relation to the role of lateralized brain function and the evolution of population level lateralization.

## Introduction

Vertebrates with laterally placed eyes typically show preferential eye use for ecological activities including scanning for potential predators (Franklin and Lima, 2001; Koboroff et al., 2008; Lustig et al., 2010; Martin et al., 2010) or searching for prey (Mench and Andrew, 1983; Robins and Rogers, 2004; Ventolini et al., 2005; Bonati et al., 2008). Processing visual information predominately through the left or right visual field has been associated with specialized function of the left and right brain (i.e., lateralized brain function; Rogers et al., 2013). Many lateralized vertebrates share a general pattern at the population level, whereby the left-brain hemisphere attends to routine behaviors (i.e., processing relevant stimuli), while the right-brain hemisphere attends to emergency responses (i.e., flight or escape responses; MacNeilage et al., 2009).

Lateralization of brain function has been associated with several cognitive advantages, including increasing neural capacity, by avoiding the duplication of functions in the two brain hemispheres (Levy, 1977). Lateralized individuals can also process information in parallel (Rogers, 2002; Rogers et al., 2004), by utilizing one hemisphere to control specific functions (Andrew, 1991; Vallortigara, 2000) and leaving the other hemisphere free to control different functions. Moreover, controlling different functions through separate hemispheres may prevent interference between conflicting responses (i.e., functional incompatibility). That is, responses evoked by stimuli that have been perceived simultaneously, whereby each stimulus demands a different response (Ingle, 1973; Vallortigara et al., 1999; Güntürkün et al., 2000; Vallortigara, 2000).

Recent studies have provided evidence of preferential eye use in invertebrate taxa, including molluscs and insects (reviewed in Frasnelli, 2013). For example, individual common octopuses, *Octopus vulgaris*, showed a significant eye preference when inspecting potential prey items (Byrne et al., 2002) and when exploring novel objects (Byrne et al., 2006). A study on European common cuttlefish, *Sepia officinalis*, showed significant left eye preference when looking for shelter (Jozet-Alves et al., 2012a). The strength of this eye preference was correlated with asymmetries in the optic lobes and vertical lobe, the primary visual processing center and multi-sensory integrative center, respectively (Jozet-Alves et al., 2012b). These studies suggest that invertebrates may predominantly use the left or right visual field to process information for specific ecological activities.

Like most coleoids (i.e., soft-bodied cephalopods), cuttlefish have laterally placed eyes and keen visual acuity. They are voracious visual predators that feed on a range of prey items (i.e., fish and crustaceans) using multiple predatory tactics including ambush predation and active hunting (Neill and Cullen, 1974; Hanlon and Messenger, 1996). Actively hunting for prey makes these soft-bodied invertebrates vulnerable to predators including dolphins, seals, sharks, and many teleost fishes as well as diving seabirds. The active predatory lifestyle of cuttlefish combined with the need to maintain a constant vigilance against predators requires effective information processing from multiple stimuli. Processing information in this way might be more efficient if each visual field is predominately used for specific functional roles, as seen in lateralized domestic chicks, *Gallus gallus*. Lateralized chicks are able to search for grain on a mixed substrate using their right eye (i.e., left brain hemisphere; Rogers, 1990), while simultaneously monitoring overhead for aerial predators using their left eye (i.e., right brain hemisphere; Rogers, 2000). This ability to search for food and monitor predators simultaneously may contribute to biological fitness. Indeed, previous studies have shown that lateralized individuals can outperform non-lateralized conspecifics in some biological circumstances (McGrew and Marchant, 1999; Güntürkün et al., 2000; Rogers et al., 2004). However, whether lateralization of brain function is also associated with cognitive advantages in invertebrate species is yet to be investigated.

In the present study, we conducted lateralization experiments on laboratory-reared European common cuttlefish to test whether they shared similar attributes of lateralization with vertebrates. As cuttlefish have been shown to favor the left eye when searching for shelter (i.e., a defensive behavior; Jozet-Alves et al., 2012a), we hypothesized that the left eye is implicated in emergency responses, while the right eye may be implicated with routine behaviors. To determine whether the left visual field is indeed associated with emergency responses, we tested whether the left eye was predominately used for scanning for potential predators (i.e., vigilant scanning). To determine whether the right visual field is associated with routine behaviors, we tested whether the right eye was predominately used for scanning for potential prey (i.e., prey attack). To investigate vigilant scanning we conducted a laboratory experiment in which individuals were introduced into a new environment and required to choose between the left or right visual field to use for scanning for potential predators. To investigate prey attack we presented individuals with a prey item in each visual field and required them to choose between attacking one prey item to the left or to the right of them. A further aim of the study was to determine whether lateralized individuals exhibited faster decision-making compared to non-lateralized individuals when they were simultaneously presented with two shrimp. We posed three main questions (1) Do cuttlefish show lateralization of vigilant scanning and prey attack? (2) If individuals exhibit visual lateralization, do cuttlefish have opposite directions of lateralization for vigilant scanning and prey attack? (3) Do lateralized cuttlefish show faster decision-making when presented with prey simultaneously?

## Materials and methods

### Animals

Ninety-three sub-adult European common cuttlefish were used in this study, ranging in age from 7 to 10 months. For experiment 1, two populations of cuttlefish were used, the first population (*N* = 10) was reared from eggs in the Grand Aquarium de Saint Malo, France (48°38′N, 2°00′W), and the second population (*N* = 83) was reared from eggs in the Marine Biological Laboratory (MBL), Marine Resources Center, Woods Hole, USA (41°31′N, 70°39′W). All the eggs were collected from the English Channel; eggs for Saint Malo were gathered along the coast of Brittany, while eggs for Woods Hole were gathered along the southern coast of England. For experiment 2, cuttlefish from experiment 1 (*N* = 72) in the MBL Marine Resources Center were re-used. Dorsal mantle lengths were measured (mean mantle length ± SEM = 44.16 ± 1.08 mm; range = 31–60 mm). Throughout these experiments, subjects were housed in groups in tanks at their respective facilities (i.e., Grand Aquarium and MBL Marine Resources Center). Tanks were supplied with a constant flow of filtered seawater (~10 L min^−1^) and maintained at a temperature of 15–17°C. Cuttlefish were maintained under daylight conditions and were fed a mixed diet of food items *ad libitum* including, thawed frozen prawn, smelt, *Osmerus eperlanus*, live eastern grass shrimp, *Palaemonetes paludosus*, and live gammarid shrimp, *Platorchestia platensis* (Krøyer, 1845). Subjects were used in several non-invasive experiments and were housed for the remainder of their life cycle (i.e., ~1 year) until they died following senescence. All applicable, international, national, and/or institutional guidelines for care and use of animals were followed. Procedures undertaken in France were approved by the regional ethical committee (Comité d'Ethique Normandie et Matiére d'Expérimentation Animale, CENOMEXA; agreement number 54). Ethical approval was not required for the experiments conducted at MBL as there are currently no ethical regulations in place for research on cephalopods in the USA.

### Test apparatus

**Figure 1:**
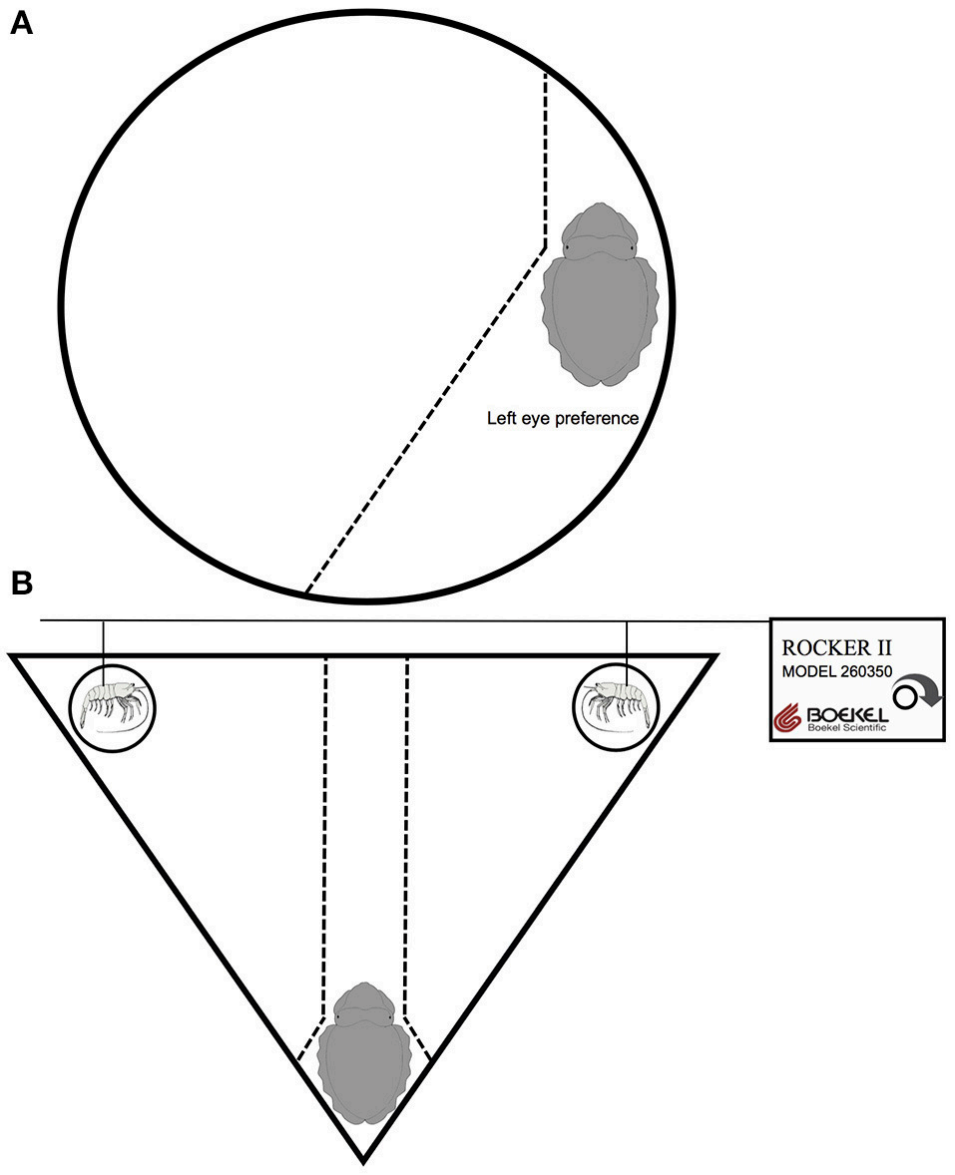
**Diagrammatic representation of the experimental apparatus for investigating (A)** anti-predatory behavior and **(B)** predatory behavior in European common cuttlefish, *Sepia officinalis*. **(A)** Depicts a cuttlefish with a left eye preference for vigilant scanning. The idealized field of view is represented by the dotted line. **(B)** Depicts a cuttlefish settled in the apex of the isosceles triangle arena and two dead shrimp, in glass vials, in opposite vertexes. Prey movement was controlled by singular metal rods attached to a horizontal pole controlled by a Boekel rocker to simulate the movement of a live shrimp. Objects are not drawn to scale.

For experiment 1, for the Saint Malo population we used a rectangular arena 800 × 300 × 400 mm (*l* × *w* × *h*), while for the Woods Hole population we used a circular arena, 260 × 90 mm (*diameter* × *h*) constructed from gray PVC (Figure 1A). A digital video camera (Sony VX-1000) was placed directly over each arena to record the vigilant scanning behavior of cuttlefish over a period of 120 min. For experiment 2, we used an isosceles triangular arena, 287 × 400 mm (*h* × *base*) constructed from gray PVC (Figure 1B). A semi-circular gray PVC barrier was placed at the apex of the arena to visually isolate subjects from the prey items and allow the cuttlefish to settle into the apex during the first phase of the test. In the two opposite vertexes, we placed a dead shrimp within a glass vial 20 × 100 mm (*diameter* × *h*). The top of each glass vial surpassed the water level in the arena, preventing chemical exchange between the prey items, and the subject. Each shrimp was supported by a metal rod, which was attached to a horizontal pole controlled by a Boekel rocker (Rocker II, model 260350, Boekel Scientific) to simulate the movement of live shrimp.

Each apparatus was illuminated by a LED strip light (I Daylight White 3528 Double Row LED, 240/m, 15 mm wide), which was placed within plastic tubing and positioned 500 mm above the center of the arena. The arenas were surrounded by black plastic walls to eliminate external cues and were supplied with a constant flow of fresh filtered seawater (95 mm deep).

### Test procedure

Experiment 1 was carried out in April 2015 in France and April 2016 in the USA. Subjects were placed individually in the arena and allowed to move freely around the apparatus. The set-up aided in determining where cuttlefish vigilance was being directed. Previous research on cuttlefish in laboratory tanks has demonstrated that they avoid open environments when they cannot bury themselves and typically align their body against an opaque surface or object (i.e., wall or rock; Alves et al., 2007). This requires them to choose which eye to orient away from the opaque arena to scan for potential predators. The panoramic field of vision of most cephalopods ensures that a large volume of water can be searched using a single visual field (Hanlon and Messenger, 1996). We are confident that this behavior is driven by the desire to scan for predators because it is typically performed when cuttlefish are introduced to open environments with predatory fish odor (i.e., gray mullet, *Mugil cephaus*; unpublished data). The orientation of individuals relative to the arena wall provided an indication of eye preference. For example, cuttlefish with a left eye preference would typically orientate the right side of their body against the arena wall and use their left eye for vigilant scanning (Figure 1A). The opposite situation held for individuals with a right eye preference. Each individual was video recorded for a 120 min period and eye preference was documented every 5 min within that period (i.e., 24 trials per individual). When individuals were not aligned against the arena wall the trial was omitted because clear eye preference was not detectable, hence some individuals participated in less than 24 trials (range: 12–24 trials).

Experiment 2 was carried out in May–June 2016. Dead eastern grass shrimp of similar size were placed within the glass vials at each vertex and the Boekel rocker was turned to the highest motion level. Cuttlefish were placed individually in the apex of the triangular arena with the semi-circular gray barrier in place to visually isolate them from the prey items. Each cuttlefish was allowed to acclimate to the new arena for a minimum of 15 min. The barrier was removed once the cuttlefish settled with the posterior end of its mantle in the point of the apex and its head facing the barrier, a position that most animals assumed within 5–25 min. Once the barrier was removed the left and right visual field of the cuttlefish were simultaneously exposed to a dead shrimp. Cuttlefish with a right eye preference would attack the dead shrimp in the right vertex and cuttlefish with a left eye preference would attack the dead shrimp in the left vertex. As soon as the subject attacked one of the glass vials, the cuttlefish was gently lifted out of the water using a small glass beaker and placed back in its home tank. If an individual did not attack either prey item within 5 min, it would be returned to its home tank and tested again the following day. This procedure was repeated once per day for each individual until they reached 10 choices. Dead eastern grass shrimp were replaced each day. We attempted to reduce uncontrolled external cues as a source of bias by rotating the arena 90° between each day of experimentation (i.e., *four* possible orientations of the apparatus, with the same number of subjects tested with each possible orientation).

### Data analysis

To determine the direction of eye preference for experiments 1 and 2, we converted the eye use data for each individual to a laterality index (LI; Bisazza et al., 2000). To calculate the LI, we used the following formula: (Number of trials where the individual used the right eye – Number of trials where the individual used the left eye)/(Total number of trials). LI is a continuous variable that ranges from −1 to +1. A left eye preference was indicated by a significantly negative value; a right eye preference was indicated by a significantly positive value. To analyse the strength of the eye preference, regardless of the direction, we also calculated the absolute value of LI. A value of 0 meant that an individual used its left and right eye equally; a value of 1 meant that an individual consistently used the same eye.

All statistical analyses were completed using R (version 2.9.0, http://www.r-project.org). We used parametric tests as well as non-parametric tests, when data did not meet the assumption of normality and homoscedasticity. To test for eye preference in each individual for both ecological activities (i.e., vigilant scanning, and prey attack), we used binomial tests. We then calculated the percentage of cuttlefish showing a left eye preference, right eye preference, or no preference for both vigilant scanning and prey attack. To compare the number of cuttlefish with a left and a right eye preference, we used a Chi-square test. To determine whether cuttlefish showed an eye preference at the population level for each ecological activity, we tested the overall LI values using a one-sample Wilcoxon test on the Saint Malo population and one-sample *t*-tests on the Woods Hole population. To test whether the mean LI differed between the two populations for vigilant scanning (i.e., Saint Malo and Woods Hole), we used exact permutation tests for independent samples. To test whether left biased cuttlefish were more strongly lateralized than right biased cuttlefish, we also used exact permutation test for independent samples for the Woods Hole population for both ecological activities. We also used one-sample Wilcoxon tests to determine eye preference for prey attack in individuals that were categorized previously as left, right or no preference for vigilant scanning. To test whether decision-making latencies during prey attack differed between lateralized and non-lateralized individuals, we used a generalized linear mixed model (GLMM). Lateralization was the predictor variable and latency (log transformed) was the dependent variable with subject as a random factor.

## Results

**Figure 2:**
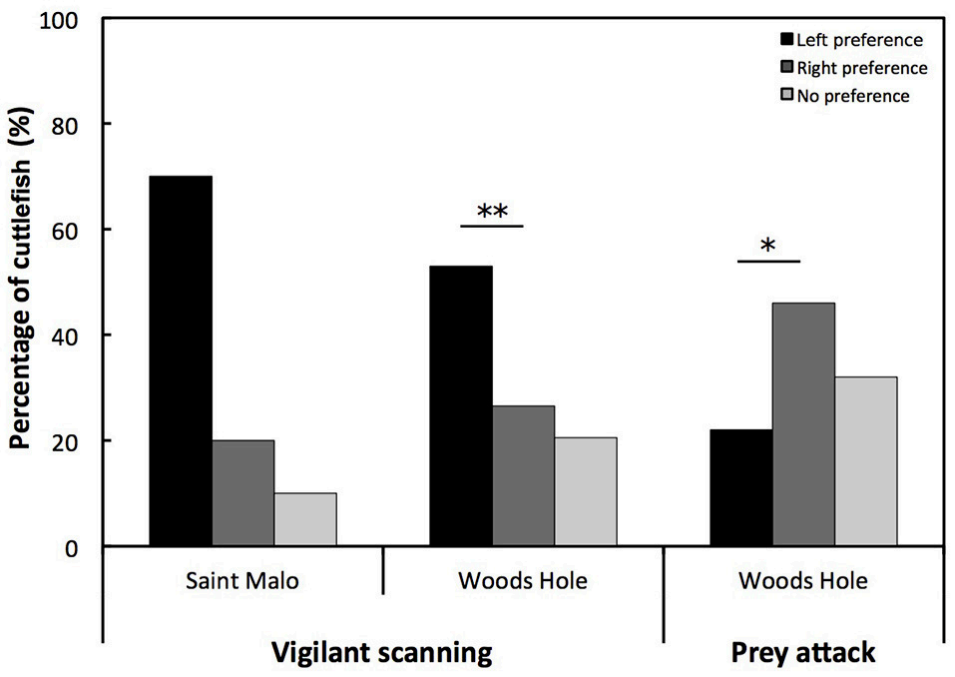
**Eye preference for vigilant scanning and prey attack**. Percentage of European common cuttlefish, *Sepia officinalis* that exhibited left eye preference, right eye preference, and no preference for vigilant scanning and prey attack. Bars marked with a solid line and accompanying asterisks represent results of a Chi-square test and signify a population level eye preference for lateralized cuttlefish. ^*^p < 0.05; ^**^p < 0.01.

Cuttlefish were categorized as left, right, or no preference (Figure 2). The number of cuttlefish with a left eye preference for vigilant scanning was higher than the number of cuttlefish with a right eye preference in the Woods Hole population [Chi-square: $\chi_{\left(1,\;\text{N}\;=\;66\right)}^{2}$ = 7.333; *p* < 0.01; Figure 2]. This was only conducted for the Woods Hole population as we were prevented from applying a Chi-square analysis on the Saint Malo population due to a low sample size. By contrast, the number of cuttlefish with a right eye preference for prey attack was higher than the number of cuttlefish with a left eye preference [Chi-square: $\chi_{\left(1,\;\text{N}\;=\;49\right)}^{2}$ = 5.898; *p* < 0.05; Figure 2].

**Figure 3:**
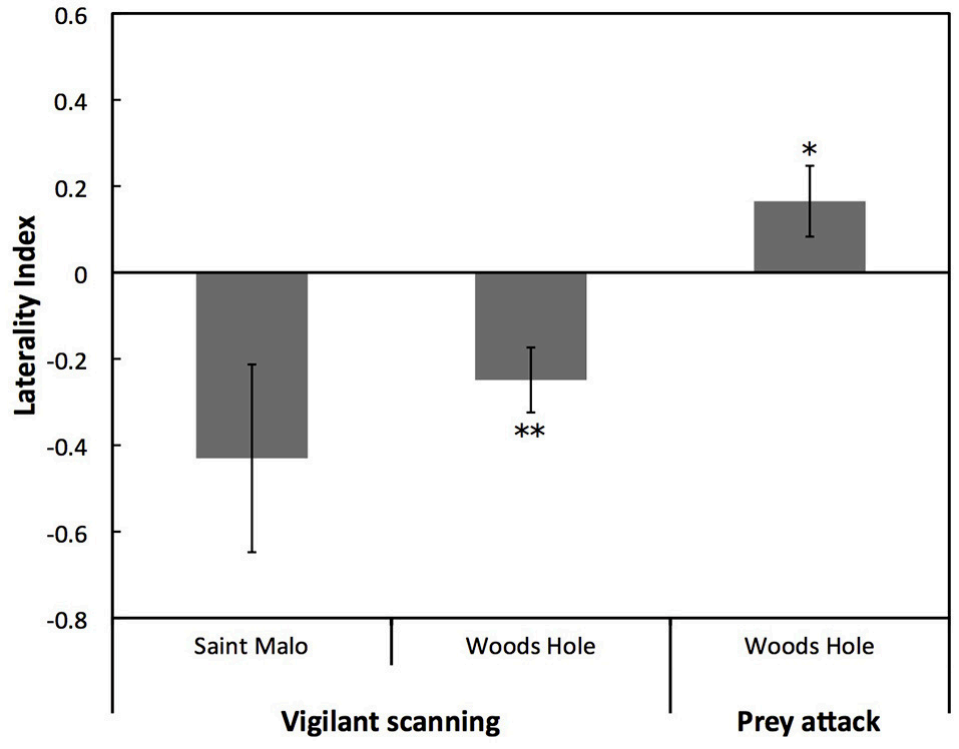
**Lateralization for vigilant scanning and prey attack**. Mean ± *SE* laterality index for vigilant scanning and prey attack in laboratory reared European common cuttlefish, *Sepia officinalis*. Negative results represent left eye preference and positive results represent right eye preference. Bars marked with asterisks represent results of one-sample *t*-tests and indicate population level eye preferences that differed significantly from 0. Significant differences between groups are indicated by a solid line and accompanying asterisks. ^*^p < 0.05; ^**^p < 0.01.

**Figure 4:**
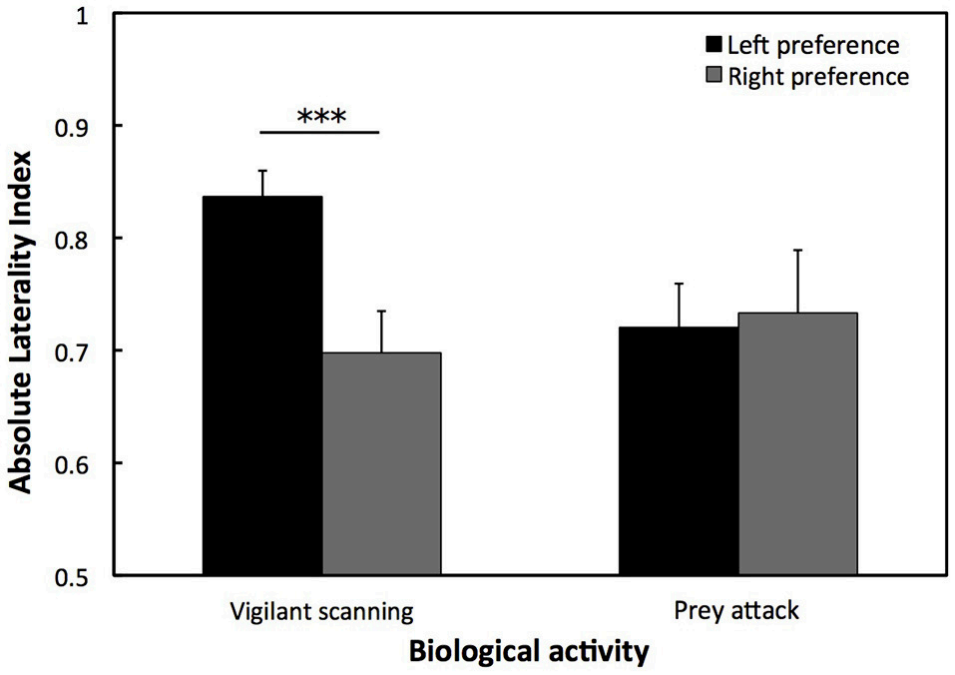
**Strength of lateralization for vigilant scanning and prey attack**. Mean ± *SE* absolute laterality index for lateralized European common cuttlefish, *Sepia officinalis* in Woods Hole. A solid line and accompanying asterisks represent results of exact permutation tests and indicates a significant difference between left and right eye preference. ^***^p < 0.001.

For the Saint Malo population, there was a statistical tendency for a population level left eye preference for vigilant scanning (*W* = 10, *p* = 0.083). Moreover, for the Woods Hole population, there was a significant population level left eye preference for vigilant scanning [*t*_(82)_ = 14.136, *p* < 0.001; Figure 3]. By contrast, there was a significant population level right eye preference for prey attack [*t*_(71)_ = 2.156, *p* < 0.05; Figure 3]. There was no significant difference of the overall LI for vigilant scanning between the two populations (exact permutation: *T* = −21.135, *p* = 0.446; Figure 3). For the Woods Hole population, comparison of absolute LI values of cuttlefish with a left and a right eye preference for vigilant scanning showed that the bias was stronger in cuttlefish displaying a left eye preference (exact permutation: *T* = 2237, *p* < 0.001; Figure 4). However, a stronger bias was not shown for prey attack: comparisons of absolute LI values of cuttlefish with a left and a right eye preference showed no significant difference (exact permutation: *T* = 1620, *p* = 0.907; Figure 4).

**Figure 5:**
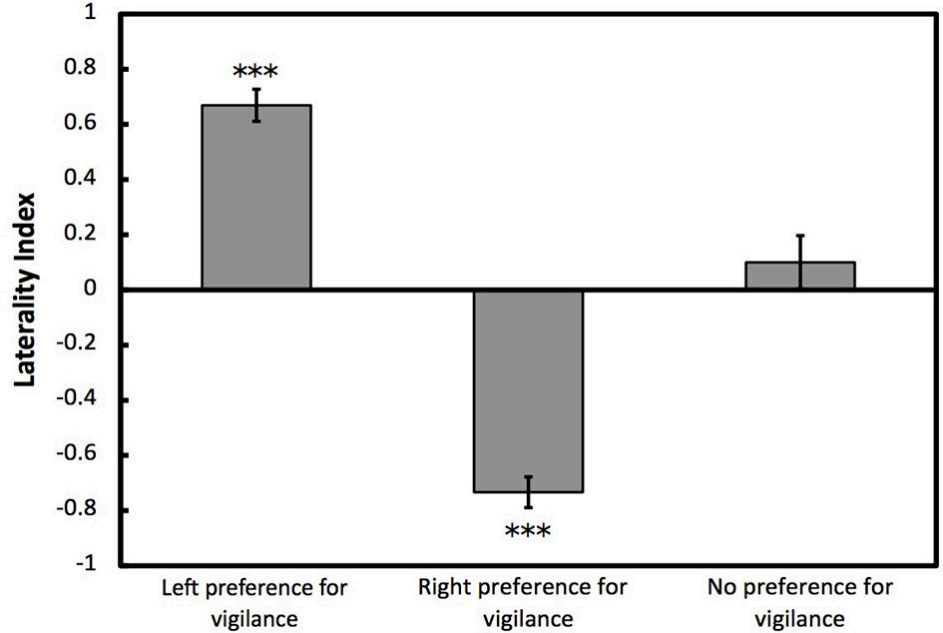
**Lateralization for prey attack for categorized individuals**. Mean ± *SE* laterality index for prey attack for European common cuttlefish, *Sepia officinalis* that had previously been categorized as left, right, or no preference for vigilant scanning. Negative results represent left eye preference for prey attack and positive results represent right eye preference prey attack. Bars marked with asterisks represent results from one-sample Wilcoxon tests. ^***^p < 0.001.

**Figure 6:**
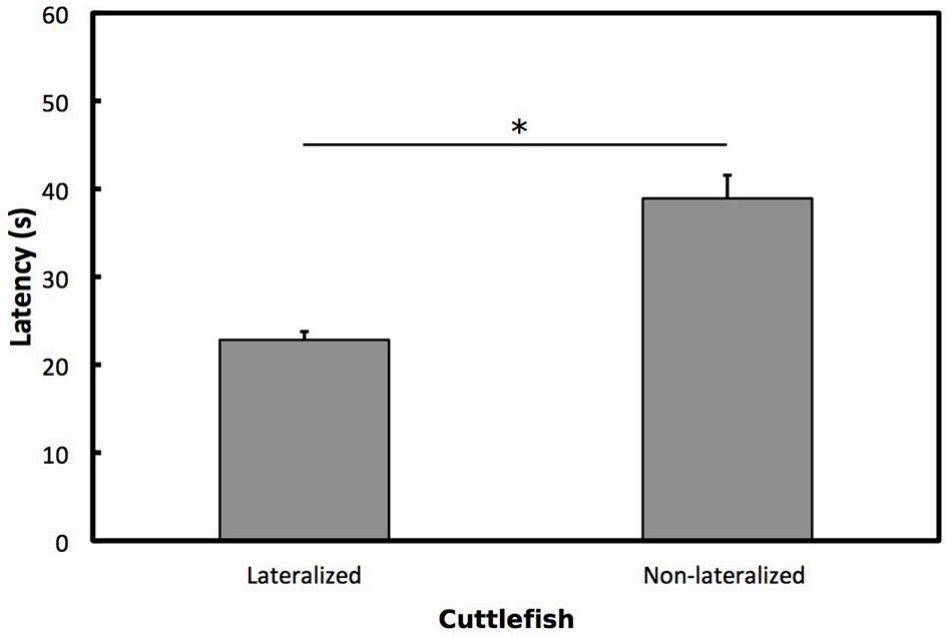
**Prey attack latencies**. Mean ± *SE* latencies for lateralized and non-lateralized European common cuttlefish, *Sepia officinalis*. Bars marked with a solid line and accompanying asterisks represent results from a GLMM and indicate a significant difference between groups. ^*^p < 0.05.

To determine whether cuttlefish showed opposite directions of lateralization for vigilant scanning and prey attack, we used one-sample Wilcoxon tests on overall LI for prey attack for individuals that had previously been categorized as left, right or no preference for vigilant scanning. Cuttlefish categorized previously as left preference for vigilant scanning showed a significant right bias for prey attack (*W* = 1.5, *p* < 0.001; Figure 5). Cuttlefish categorized previously as right preference for vigilant scanning showed a significant left bias for prey attack (*W* = 780, *p* < 0.001). Cuttlefish that were categorized previously as having no preference for vigilant scanning did not show any eye preference for prey attack (*W* = 15, *p* = 0.395; Figure 5). Lateralized cuttlefish (i.e., individuals exhibiting either left or right preference for prey attack) attacked shrimp faster than non-lateralized cuttlefish (GLMM: χ^2^ = 5.859, *p* < 0.05; Figure 6).

## Discussion

Our study provides behavioral evidence of lateralization of brain function in the European common cuttlefish. We found that most cuttlefish exhibited lateralization for vigilant scanning and prey attack. Cuttlefish were lateralized at the population level; that is, most cuttlefish were significantly more likely to favor the left visual field to scan for potential predators. This pattern is comparable to previous studies on lateralization of anti-predatory behavior, revealing that cuttlefish exhibit a left eye preference when seeking shelter (Jozet-Alves et al., 2012b). Our data also demonstrate that cuttlefish were significantly more likely to favor the right visual field for prey attack. Furthermore, cuttlefish that were leftward directed for vigilant scanning were predominately rightward directed for attacking prey. The opposite situation held for individuals with a rightward preference for vigilant scanning. This indicates that cuttlefish have opposite directions of lateralization for vigilant scanning and prey attack. Lateralized individuals also showed faster decision-making when presented with prey simultaneously, suggesting that lateralized cuttlefish may have a cognitive advantage over non-lateralized conspecifics.

The behavioral evidence for lateralization of eye use in cuttlefish suggests that there is associated specialized brain function. Our results demonstrate that cuttlefish have opposite directions of lateralization for emergency responses (i.e., vigilant scanning and shelter) and routine behaviors (i.e., prey attack). Behavioral lateralization in vertebrate taxa is considered to be a consequence of specialized function of the left and right brain hemisphere. However, cephalopods do not have obvious left and right brain hemispheres, but they do exhibit paired structures of the central nervous system. These paired structures include the optic lobes, which are implicated *inter alia* in visual processing and located behind the eyes (Nixon and Young, 2003). Previous research has shown that individual cuttlefish exhibit anatomical asymmetries in the size of the left and right optic lobes and these asymmetries are correlated with behavioral lateralization (Jozet-Alves et al., 2012b). Interestingly, a correlation was also found between an unpaired structure, the vertical lobe, and behavioral lateralization. Cuttlefish with a larger right optic lobe and a vertical lobe with an engorged right side showed a stronger left-turning bias when seeking shelter (Jozet-Alves et al., 2012b). Furthermore, only one side of the cortex of the vertical lobe was activated when the corresponding eye was exposed to light (unpublished data). There are two plausible explanations for these correlations between behavioral and brain asymmetries. First, one part of the brain may be more dominate than its counterpart, which may explain why cuttlefish favored their left eye when searching for shelter (Jozet-Alves et al., 2012a). Second, the brain is specialized, whereby each side of the brain predominately processes information for specific ecological activities. Our results support the latter notion as cuttlefish in our study used specific visual fields for vigilant scanning and prey attack. The use of both left and right visual fields for particular ecological activities suggests that one part of the brain is not dominate over the other, rather there appears to be functional specialization of each optic lobe. Anatomical brain asymmetry has also been observed in another cephalopod species, the deep-sea squid, *Histioteuthis* (Wentworth and Muntz, 1989). In this species, individuals possess a large left optic lobe, used to look upwards in the water column to potentially detect predators. Conversely, the right optic lobe is considerably smaller and orients downwards to potentially search for prey.

In our study, most individuals showed a similar direction of bias, significantly favoring the left visual field for vigilant scanning and the right visual field for prey attack. This bias indicates that cuttlefish exhibit population level lateralization for these ecological activities. Although brain lateralization is thought to provide benefits such as performing simultaneous tasks more efficiently (i.e., vigilance and foraging; Dadda and Bisazza, 2006), lateralization does not need to be expressed at the population level to attain such benefits. In fact, lateralization at the population level may have some drawbacks, because it makes the behavior of each individual more predictable to other animals (i.e., potential predators or prey; Ghirlanda and Vallortigara, 2004). For example, if cuttlefish predominately used the left visual field to scan for predators, a predator could learn to exploit this bias and always attack from the right. This disadvantage would not occur if the direction of lateralization varied from one individual cuttlefish to another. The social constraint hypothesis has been proposed as a framework for understanding the reason animals exhibit population level biases (Ghirlanda and Vallortigara, 2004). In a prey-predator context, the hypothesis suggests that population level lateralization may have evolved due to social pressures that require individuals to align the direction of their bias with the direction of the other individuals of the group (Vallortigara and Rogers, 2005). This hypothesis has been supported by studies on turning biases of fish when escaping from predators. For example, many shoaling species of fish exhibit population level lateralization for turning behavior, whereas most non-shoaling fish species only show lateralization at the individual level (Bisazza et al., 2000).

Social constraints may influence whether biases occur at the individual level or the population level in cephalopods. Octopus and cuttlefish vary in their degree of sociality, ranging from solitary to aggregating species. Interestingly, solitary common octopuses show significant eye preference when presented with a crab, yet show no population level bias (Byrne et al., 2002, 2004). However, European common cuttlefish, which form loose aggregations (i.e., 3–8 individuals) briefly during reproduction, show a weak population level eye preference (e.g., 55–60%). These lateralization differences across various cephalopod species deserve further exploration, particularly in studies of brain function.

Comparisons between lateralized cuttlefish that exhibited a left or right eye preference for vigilant scanning showed that the strength of lateralization was stronger in leftward directed cuttlefish. Despite the prevalence of brain lateralization across taxa, there is considerable intraspecific variation in the strength of lateralization. Previous research has shown that in humans, right-handers are more consistent in their hand preference for various tasks compared to left-handers (Oldfield, 1971). In these cases, the bias of the more strongly lateralized individuals is consistent with the population level bias. However, the results obtained from humans are difficult to interpret, as there are potential cultural factors that influence handedness. For this reason, cuttlefish may be a useful model to explore why individuals that exhibit population level biases are more strongly lateralized than their counterparts that have an opposite pattern of specialization.

Our study also showed that when cuttlefish were simultaneously presented with two shrimp, one visible in the left visual field and the other in the right visual field, lateralized individuals exhibited faster decision-making compared to non-lateralized individuals. That is, lateralized cuttlefish showed shorter latencies to prey attack than non-lateralized conspecifics. Lateralized cuttlefish may have an advantage because information is prioritized by one visual field when searching for prey. This is one of the few examples showing that lateralized individuals could have a cognitive advantage over non-lateralized individuals in an invertebrate species (but see also Pascual et al., 2004). Our results provide further evidence that brain lateralization plays an important role in cognitive function and suggests that laterality may lead to fitness consequences for organisms in their natural environments. However, further exploration is needed to determine whether lateralized cuttlefish are more efficient at performing two tasks simultaneously than non-lateralized conspecifics. This can be tested using a dual-task design, to determine whether strength of lateralization is associated with the ability to scan for predators and search for prey simultaneously.

In conclusion, this study demonstrates that cuttlefish share similar attributes of lateralization with many vertebrate species. In fact, the pattern observed in cuttlefish is comparable to the pattern observed in most vertebrate taxa, whereby the left visual field plays a predominate role in emergency responses and the right visual field plays a predominate role in routine behaviors. This suggests that there are strong selective pressures driving general patterns of lateralization across diverse groups of animals. To our knowledge, our study provides the first evidence of lateralization homology between invertebrates and vertebrates (i.e., emergency responses and routine behaviors processed by different parts of the brain).

## Author contributions

AS contributed to the conception of the study, the experimental design, the data collection, the statistical analysis, and wrote the manuscript. RH contributed to the conception of the study, the experimental design, the data collection, the analysis and interpretation of the results, and assisted with the writing of the manuscript. AB contributed to the conception of the study, the experimental design, the data collection, the presentation of the results, and assisted with the writing of the manuscript. CJ-A contributed to the conception of the study, the experimental design, the data collection, the analysis and interpretation of the results, and assisted with the writing of the manuscript.

## Funding

This work was supported by a post-doctoral study grant from the Fyssen Foundation to AS, and by a research grant “Sélavie” from the Fyssen Foundation to CJ-A. The Sholley Foundation provided partial support for the research in Woods Hole.

### Conflict of interest statement

The authors declare that the research was conducted in the absence of any commercial or financial relationships that could be construed as a potential conflict of interest.
